# Supplementary material for: CDC20-Mediated hnRNPU Ubiquitination Regulates Chromatin Condensation and Anti-Cancer Drug Response
Source: Cancers (Basel). 2022 Jul 31;14(15):3732. doi: 10.3390/cancers14153732 (PMC9367339; doi:10.3390/cancers14153732)
Supplement: Supplementary file 1 [file cancers-14-03732-s001.zip › cancers-1808141-supplementary.pdf]

Supplementary Materials: CDC20-Mediated hnRNPU Ubiquitination Regulates Chromatin Condensation and Anti-Cancer Drug Response

Cindy Wavelet-Vermuse, Olena Odnokoz, Yifan Xue, Xinghua Lu, Massimo Cristofanilli and Yong Wan

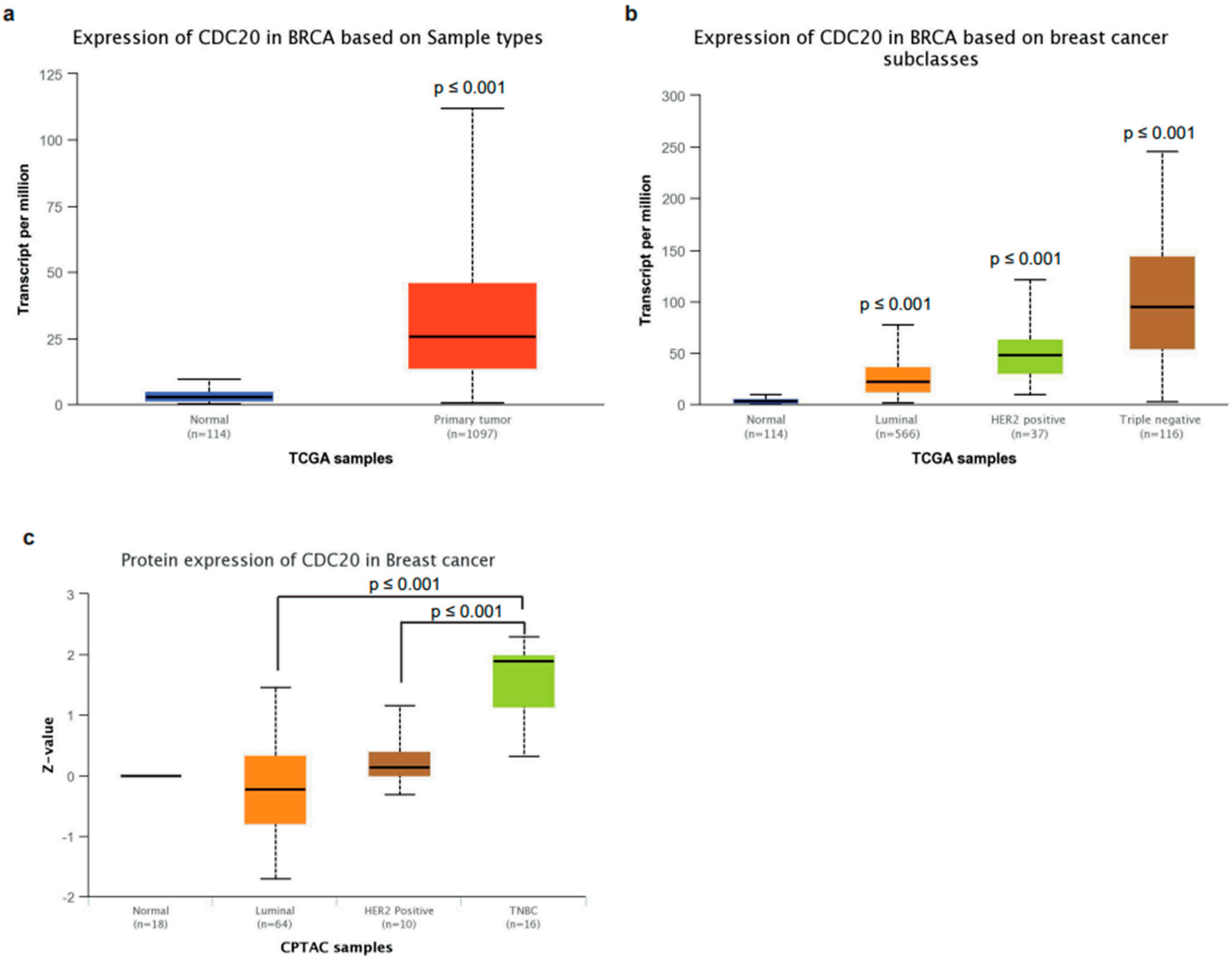

Figure S1. (**a**) Box plots showing relative expression of CDC20 mRNA in breast cancer and normal tissues. The mRNA expression levels were normalized as transcripts per million reads. (**b**) Box plots showing relative expression of CDC20 mRNA among breast cancer subtypes. (**c**) Box plots showing relative expression of CDC20 protein in different breast cancer subtypes. The transcriptome and proteome data were obtained from TCGA and CPTAC databases, respectively, and box plots were generated using the UALCAN web resource.

22h docetaxel

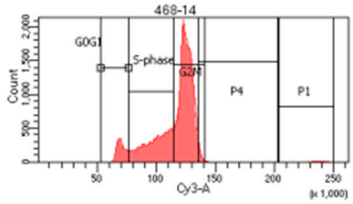

Tube: 14

Population	#Events	%Parent	%Total
All Events	49,316	####	100.0
Singlet gate	44,020	89.3	89.3
G0G1	3,365	7.6	6.8
S-phase	12,610	28.6	25.6
G2M	26,443	60.1	53.6
P1	755	1.7	1.5
P4	1,218	2.8	2.5
P2	44,727	90.7	90.7
P3	42,839	86.9	86.9

23h docetaxel

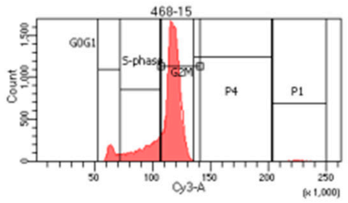

Tube: 15

Population	#Events	%Parent	%Total
All Events	35,918	####	100.0
Singlet gate	29,934	83.3	83.3
G0G1	1,872	6.3	5.2
S-phase	5,780	19.3	16.1
G2M	20,972	70.1	58.4
P1	513	1.7	1.4
P4	382	1.3	1.1
P2	29,651	82.6	82.6
P3	29,235	81.4	81.4

26h docetaxel

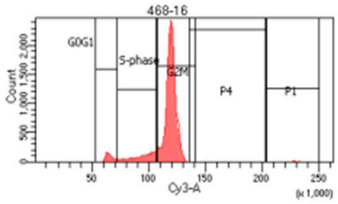

Tube: 16

Population	#Events	%Parent	%Total
All Events	34,626	####	100.0
Singlet gate	28,277	81.7	81.7
G0G1	1,444	5.1	4.2
S-phase	3,777	13.4	10.9
G2M	21,812	77.1	63.0
P1	559	2.0	1.6
P4	340	1.2	1.0
P2	28,071	81.1	81.1
P3	27,552	79.6	79.6

32h docetaxel

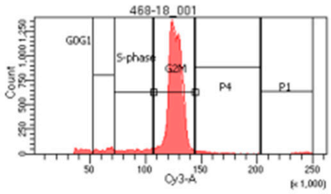

Tube: 18_001

Population	#Events	%Parent	%Total
All Events	42,364	####	100.0
Singlet gate	24,834	58.6	58.6
G0G1	1,036	4.2	2.4
S-phase	1,676	6.7	4.0
G2M	19,347	77.9	45.7
P1	645	2.6	1.5
P4	1,173	4.7	2.8
P2	23,750	56.1	56.1
P3	23,736	56.0	56.0

44h docetaxel

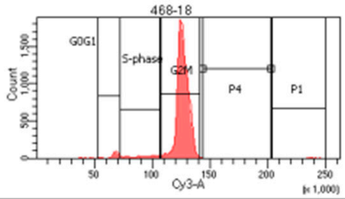

Tube: 18

Population	#Events	%Parent	%Total
All Events	33,350	####	100.0
Singlet gate	23,244	69.7	69.7
G0G1	897	3.9	2.7
S-phase	1,376	5.9	4.1
G2M	19,738	84.9	59.2
P1	445	1.9	1.3
P4	324	1.4	1.0
P2	23,090	69.2	69.2
P3	22,713	68.1	68.1

68h docetaxel

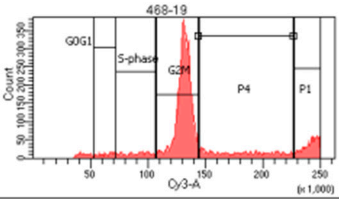

Tube: 19

Population	#Events	%Parent	%Total
All Events	11,680	####	100.0
Singlet gate	8,076	69.1	69.1
G0G1	277	3.4	2.4
S-phase	545	6.7	4.7
G2M	4,717	58.4	40.4
P1	868	10.7	7.4
P4	1,429	17.7	12.2
P2	7,547	64.6	64.6
P3	6,165	52.8	52.8

2h hesperadin

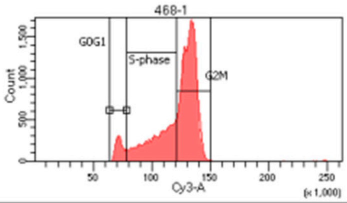

Tube: 1

Population	#Events	%Parent	%Total
All Events	47,481	####	100.0
Singlet gate	40,731	85.8	85.8
G0G1	2,670	6.6	5.6
S-phase	12,562	30.8	26.5
G2M	24,376	59.8	51.3
P2	41,001	86.4	86.4
P3	39,760	83.7	83.7

3h hesperadin

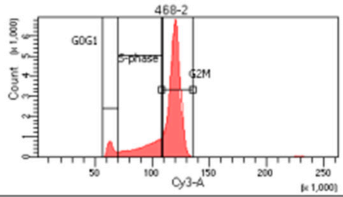

Tube: 2

Population	#Events	%Parent	%Total
All Events	122,131	####	100.0
Singlet gate	101,806	83.4	83.4
G0G1	5,901	5.8	4.8
S-phase	22,074	21.7	18.1
G2M	71,235	70.0	58.3
P2	101,216	82.9	82.9
P3	99,502	81.5	81.5

6h hesperadin

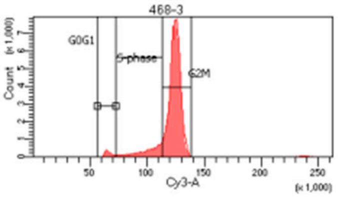

Tube: 3

Population	#Events	%Parent	%Total
All Events	115,964	####	100.0
Singlet gate	100,957	87.1	87.1
G0G1	3,471	3.4	3.0
S-phase	14,806	14.7	12.8
G2M	78,981	78.2	68.1
P2	99,698	86.0	86.0
P3	98,146	84.6	84.6

12h hesperadin

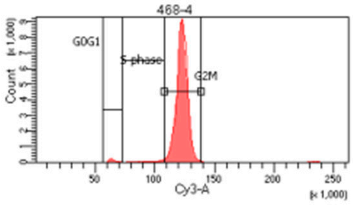

Tube: 4

Population	#Events	%Parent	%Total
All Events	122,512	####	100.0
Singlet gate	101,875	83.2	83.2
G0G1	2,479	2.4	2.0
S-phase	4,165	4.1	3.4
G2M	90,305	88.6	73.7
P2	96,565	78.8	78.8
P3	99,191	81.0	81.0

24h hesperadin

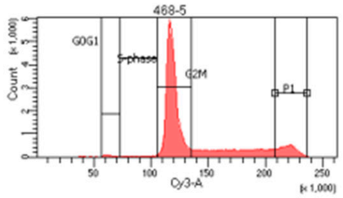

Tube: 5

Population	#Events	%Parent	%Total
All Events	125,635	####	100.0
Singlet gate	101,596	80.9	80.9
G0G1	1,575	1.6	1.3
S-phase	2,500	2.5	2.0
G2M	59,860	58.9	47.6
P1	10,665	10.5	8.5
P2	94,776	75.4	75.4
P3	73,532	58.5	58.5

48h hesperadin

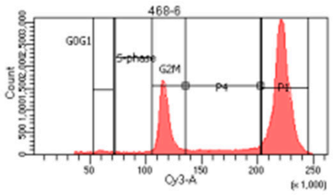

Tube: 6

Population	#Events	%Parent	%Total
All Events	139,500	####	100.0
Singlet gate	79,966	57.3	57.3
G0G1	1,597	2.0	1.1
S-phase	2,491	3.1	1.8
G2M	18,272	22.8	13.1
P1	47,421	59.3	34.0
P4	8,301	10.4	6.0
P2	43,546	31.2	31.2
P3	26,463	19.0	19.0

Figure S2. Representative flow cytometry histograms. MDA-MB-468 cells were pre-treated with 100 nM of docetaxel for 20 h. Pre-treated cells were cultured with either 50 nM of hesperadin or DMSO for 2, 3, 6, 12, 24, and 48 h in the presence of docetaxel. Flow cytometry analysis of two biological replicates after propidium iodide staining was performed to determine the percentage of cells in the G1, S, and G2/M phases.

a

DMSO	*3h vs 2h*	*6h vs 2h*	*12h vs 2h*	*24h vs 2h*	*48h vs 2h*
G0/G1	ns	ns	ns	ns	ns
S	ns	p < 0.05	p < 0.05	p < 0.05	p < 0.05
G2/M	ns	p < 0.001	p < 0.001	p < 0.001	p < 0.01
4N DNA	ns	ns	ns	ns	p < 0.01

Hesperadin	*3h vs 2h*	*6h vs 2h*	*12h vs 2h*	*24h vs 2h*	*48h vs 2h*
G0/G1	p < 0.05	p < 0.001	p < 0.001	p < 0.001	p < 0.001
S	ns	ns	ns	ns	ns
G2/M	ns	ns	p < 0.05	ns	p < 0.05
4N DNA	ns	ns	ns	p < 0.01	p < 0.01

**Hesperadin compared to DMSO group**						
	2h	*3h*	*6h*	*12h*	*24h*	*48h*
G0/G1	ns	ns	p < 0.01	p < 0.01	ns	p < 0.01
S	ns	ns	ns	ns	ns	p < 0.05
G2/M	ns	ns	ns	ns	p < 0.001	p < 0.01
4N DNA	ns	ns	ns	ns	ns	p < 0.01

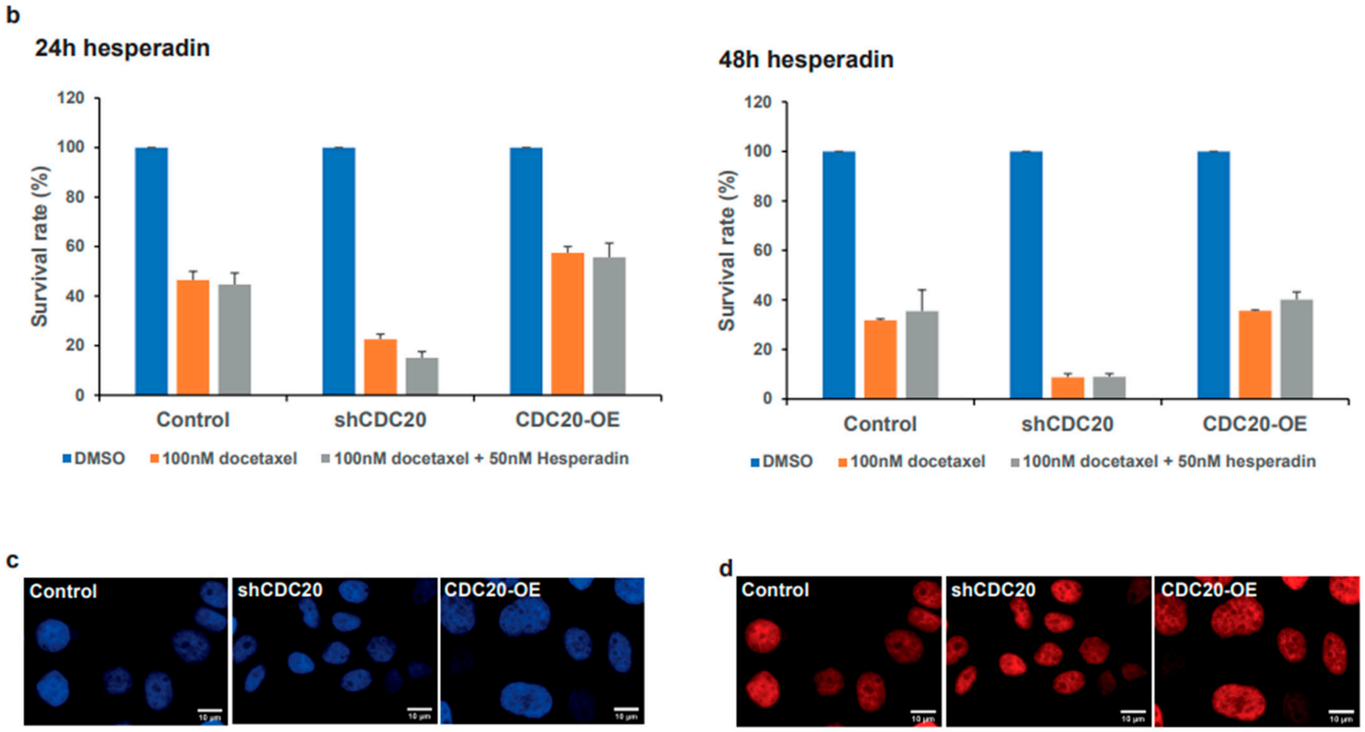

Figure S3. (**a**) Statistical analysis for the flow cytometry analysis in figure 2b. (**b**) MDA-MB-468 cells control, expressing CDC20 knockdown or overexpressing CDC20 were pre-treated with 100 nM of docetaxel for 20 h. Pre-treated cells were cultured with either 50 nM of hesperadin or DMSO for 24 and 48 h in the presence of docetaxel. The percentage of surviving cells was measured by CCK-8 cell survival assays. (**c**) Representative images for figure 2c. (**d**) Representative images for figure 2d.

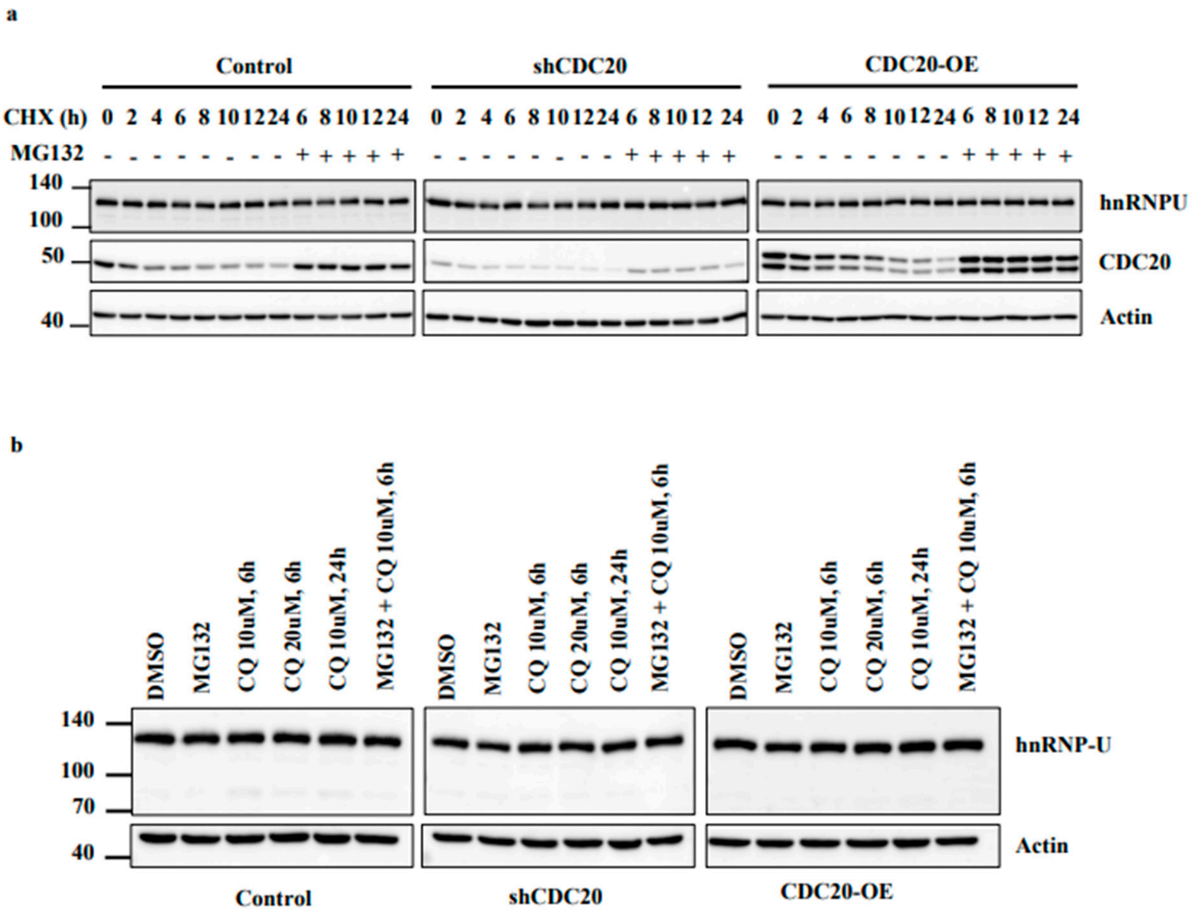

Figure S4. (**a**) Pulse-chase analysis for MDA-MB-468 cells control, expressing CDC20 knockdown or overexpressing CDC20. Cells were treated with 100 μg/mL cycloheximide (CHX) at the indicated time points, with or without MG-132. hnRNPU levels were measured by immunoblotting. Actin was used as a loading control. (**b**) MDA-MB-468 cells control, expressing CDC20 knockdown or overexpressing CDC20 were treated with MG132 or/and chloroquine (CQ). hnRNPU levels were measured by immunoblotting. Actin was used as a loading control.

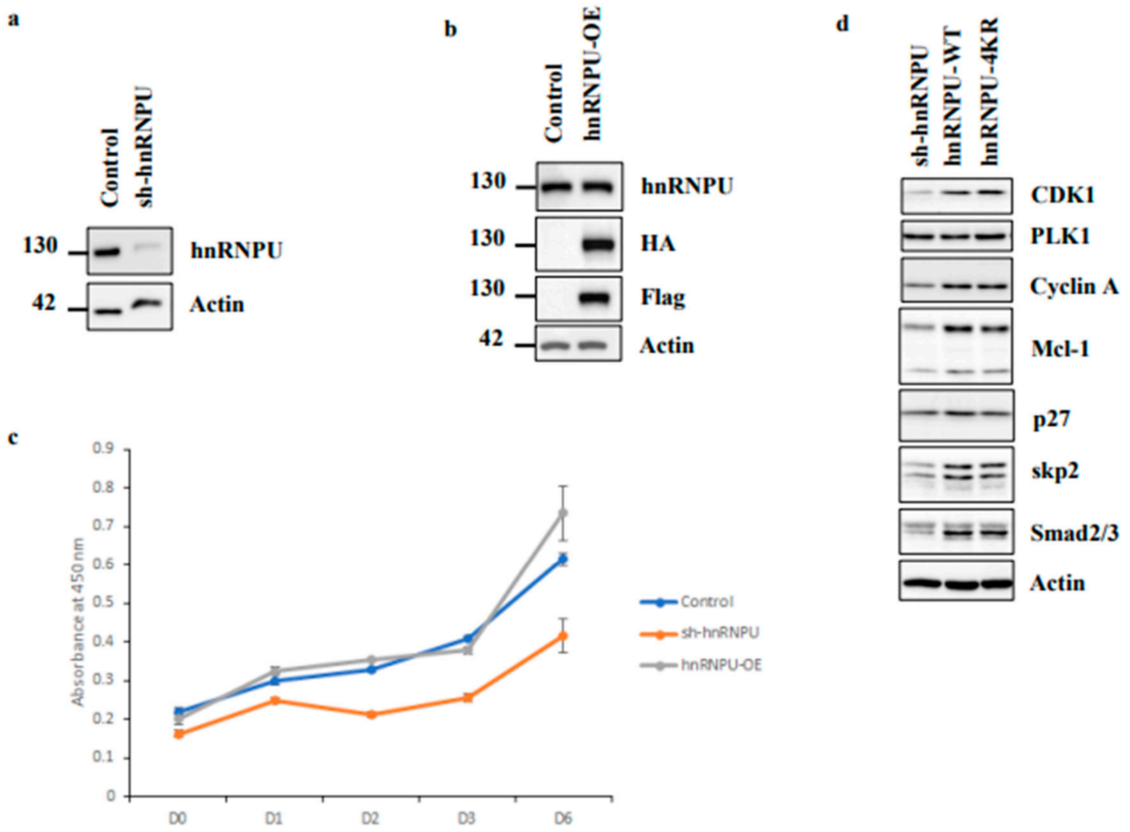

Figure S5. (**a**) MDA-MB-468 cells were transduced with either scramble control shRNA or hnRNPU shRNA and processed by immunoblotting. (**b**) MDA-MB-468 cells were transduced with pHAGE-hnRNPU plasmid to overexpress hnRNPU. The levels of hnRNPU were measured by immunoblotting. (**c**) Cell proliferation of MDA-MB-468 control cells, expressing hnRNPU knockdown or overexpression was measured using CCK8. (d) MDA-MB-468 cells expressing hnRNPU knockdown were transduced with hnRNPU-WT or hnRNPU-4KR to rescue hnRNPU. The levels of CDK1, PLK1, Cyclin A, Mcl-1, p27, skp2, and Smad2/3 were measured by immunoblotting.

Figure 1a:

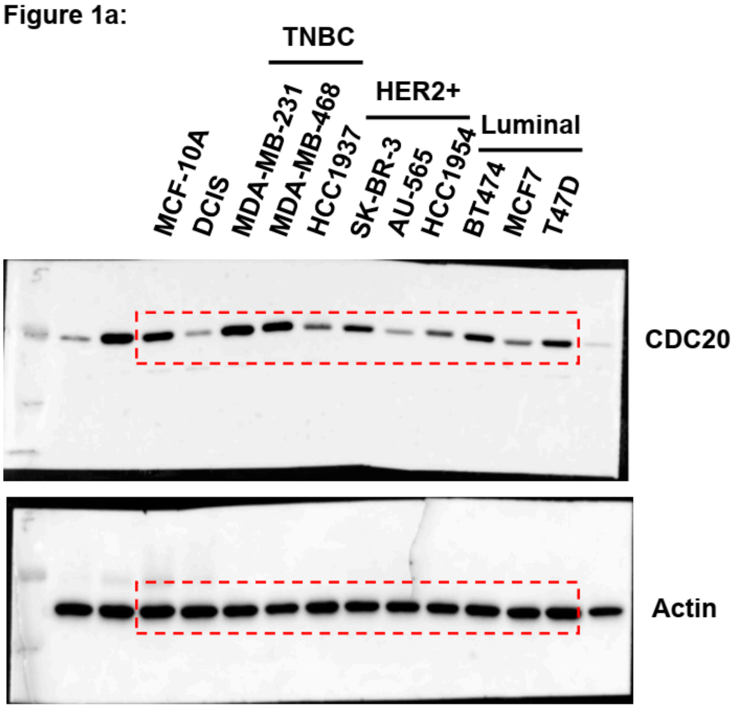

Figure 1c:

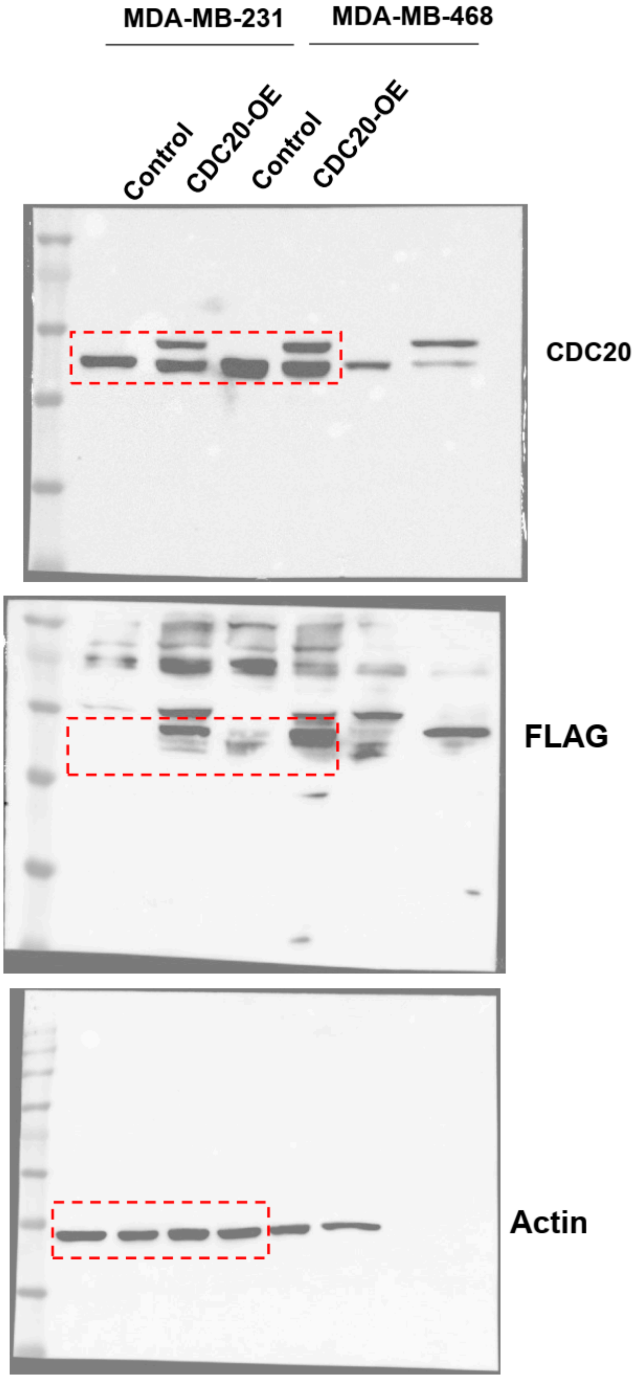

Figure 1c:

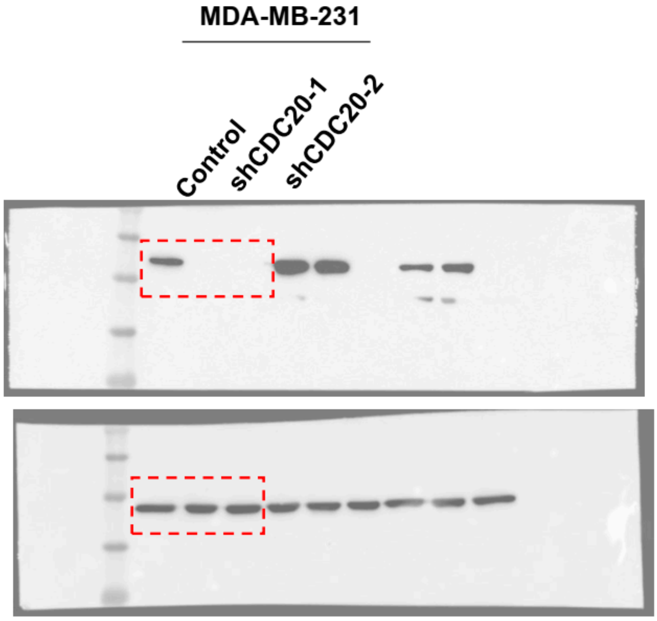

Figure 2a:

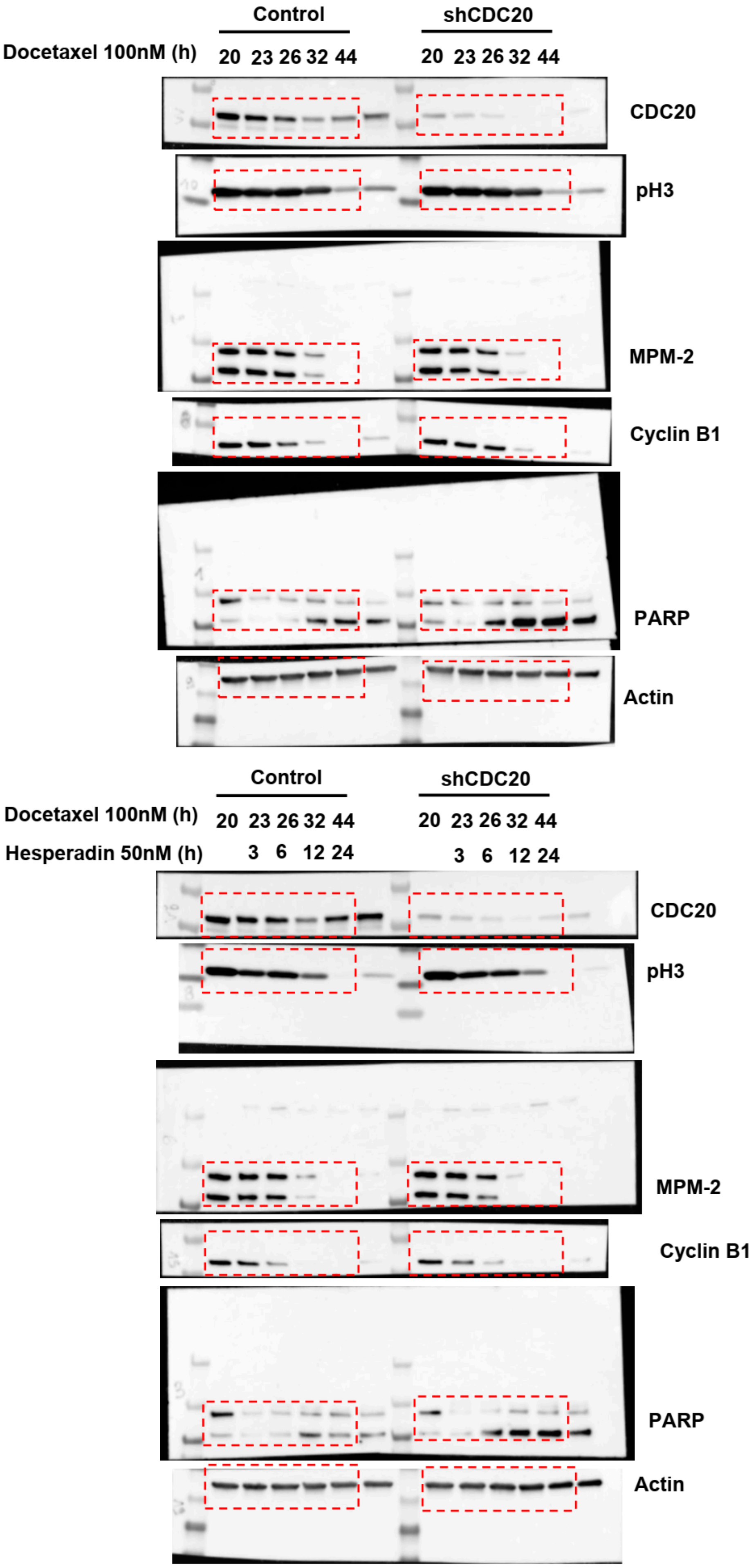

Control
shCDC20
Docetaxel 100nM (h)
20 23 26 32 44
20 23 26 32 44
CDC20
pH3
MPM-2
Cyclin B1
PARP
Actin
Control
shCDC20
Docetaxel 100nM (h)
20 23 26 32 44
20 23 26 32 44
Hesperadin 50nM (h)
3 6 12 24
3 6 12 24
CDC20
pH3
MPM-2
Cyclin B1
PARP
Actin

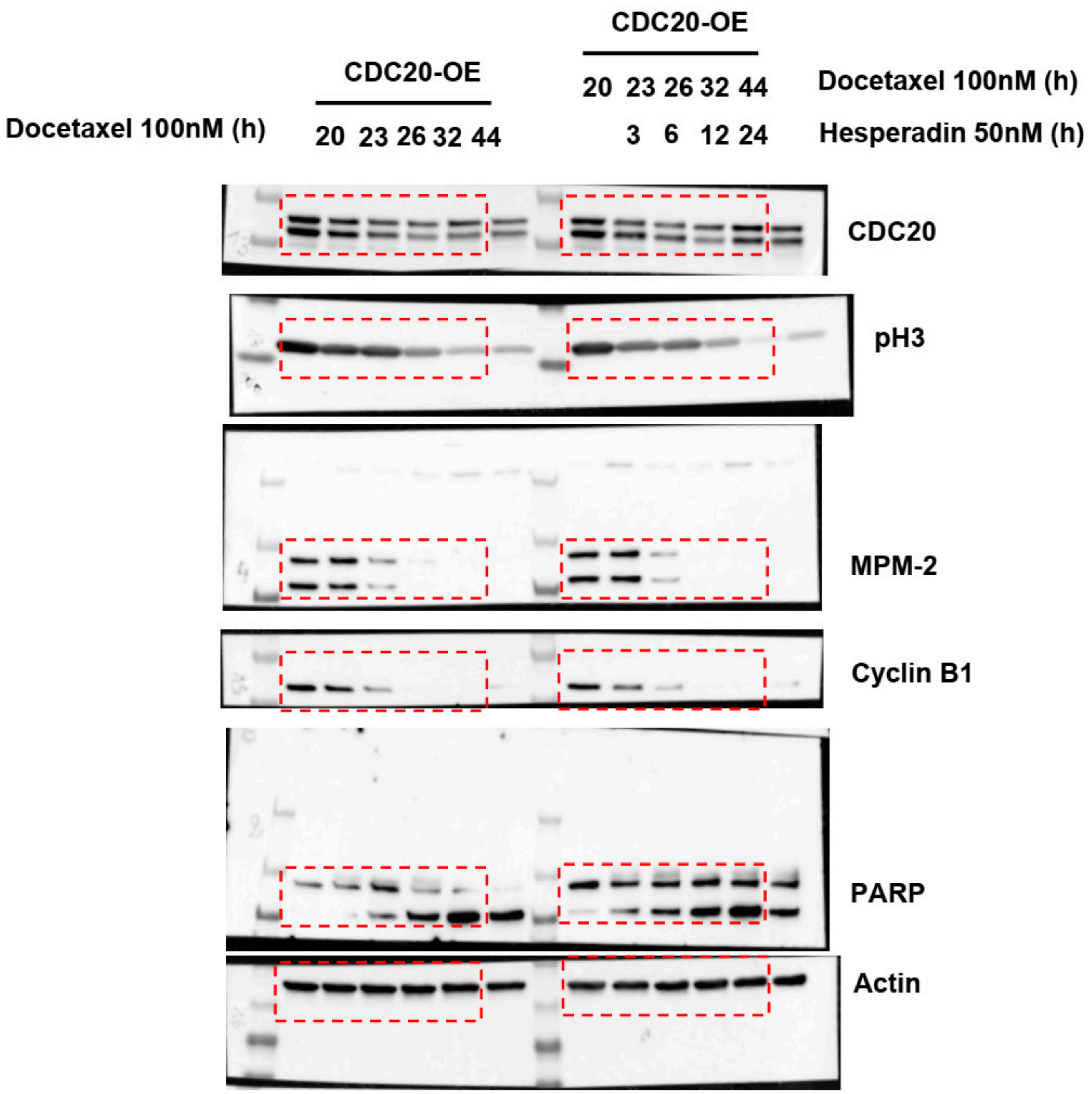
CDC20-OE
CDC20-OE
Docetaxel 100nM (h)
20 23 26 32 44
20 23 26 32 44
Docetaxel 100nM (h)
3 6 12 24
Hesperadin 50nM (h)
CDC20
pH3
MPM-2
Cyclin B1
PARP
Actin

Figure 3b:

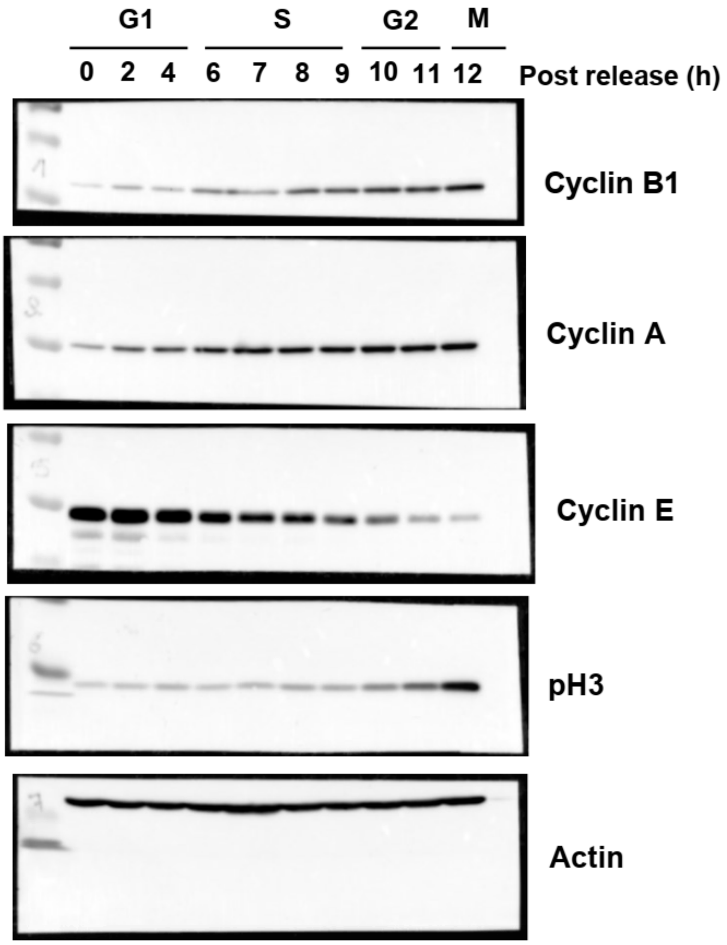

Figure 3c:

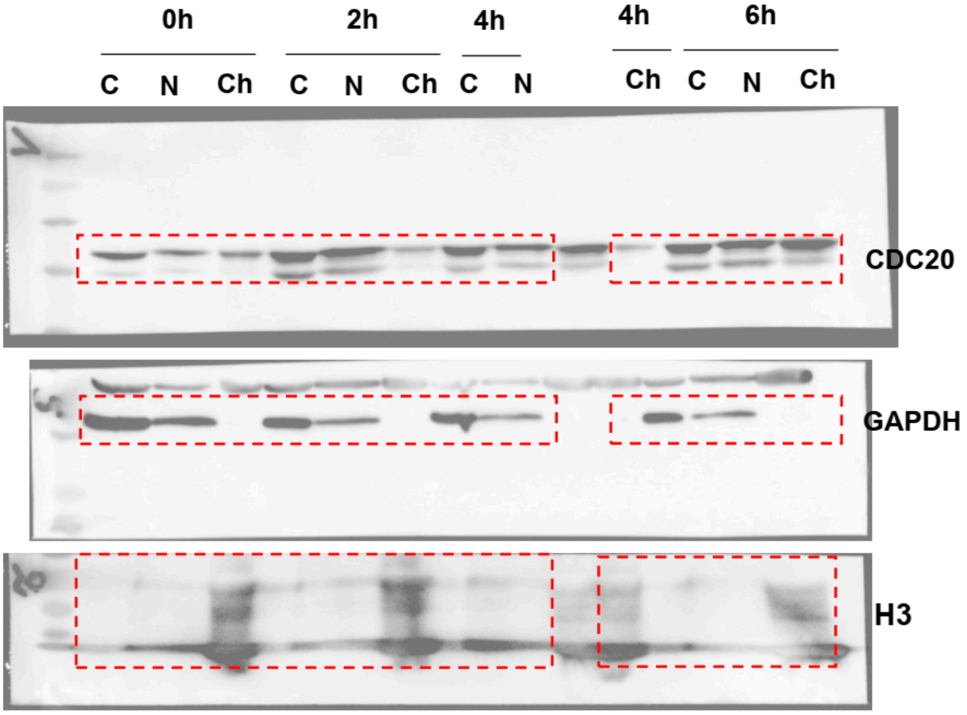

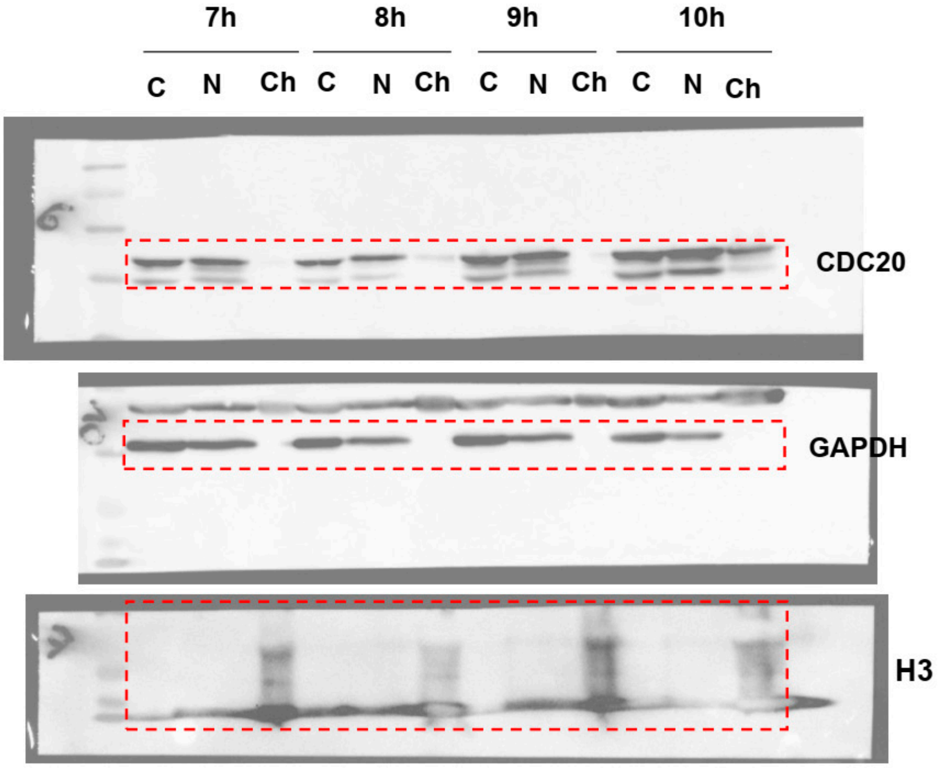
7h
8h
9h
10h
C N Ch C N Ch C N Ch C N Ch
CDC20
GAPDH
H3

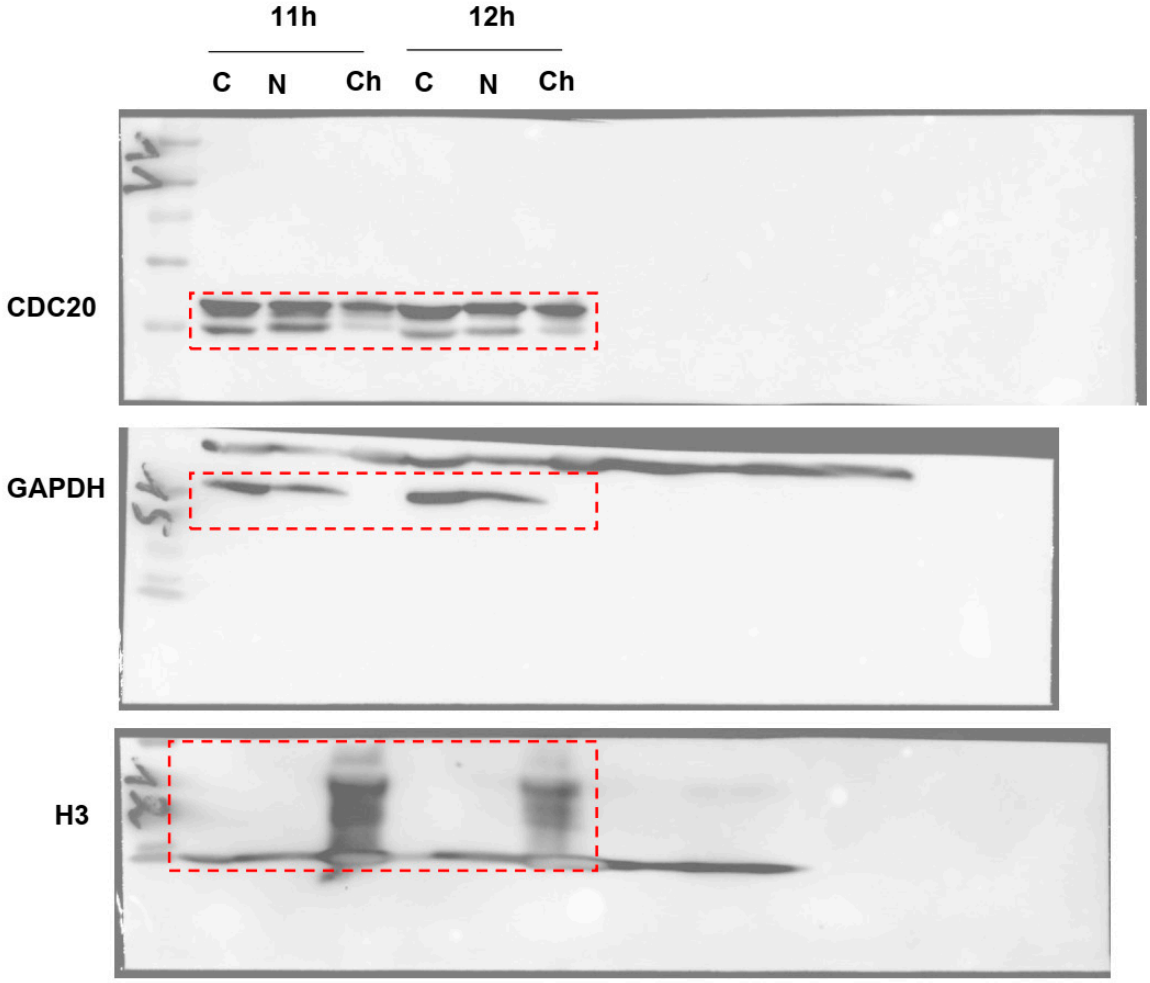
11h
12h
C N Ch C N Ch
CDC20
GAPDH
H3

Figure 3d:

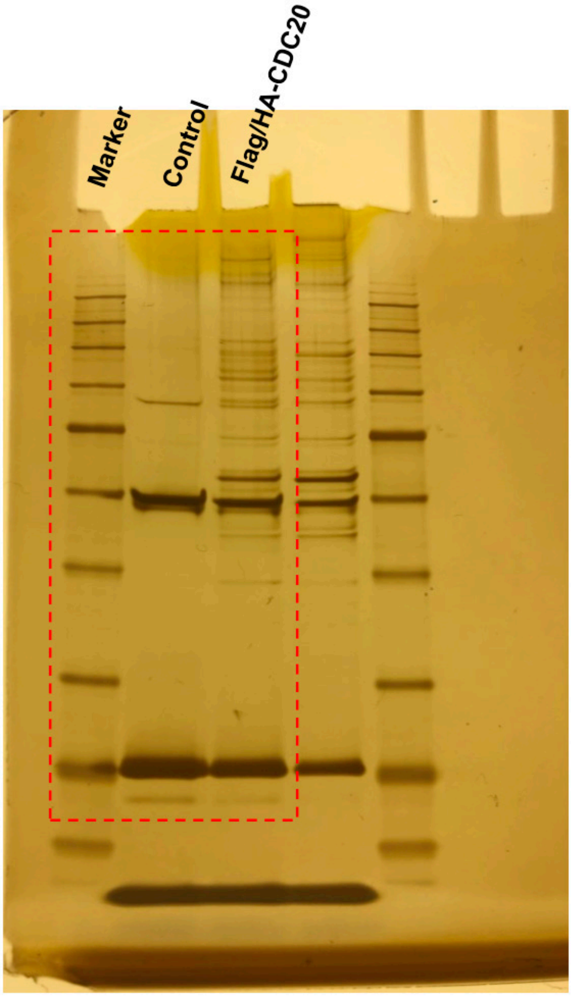

Figure 3f:

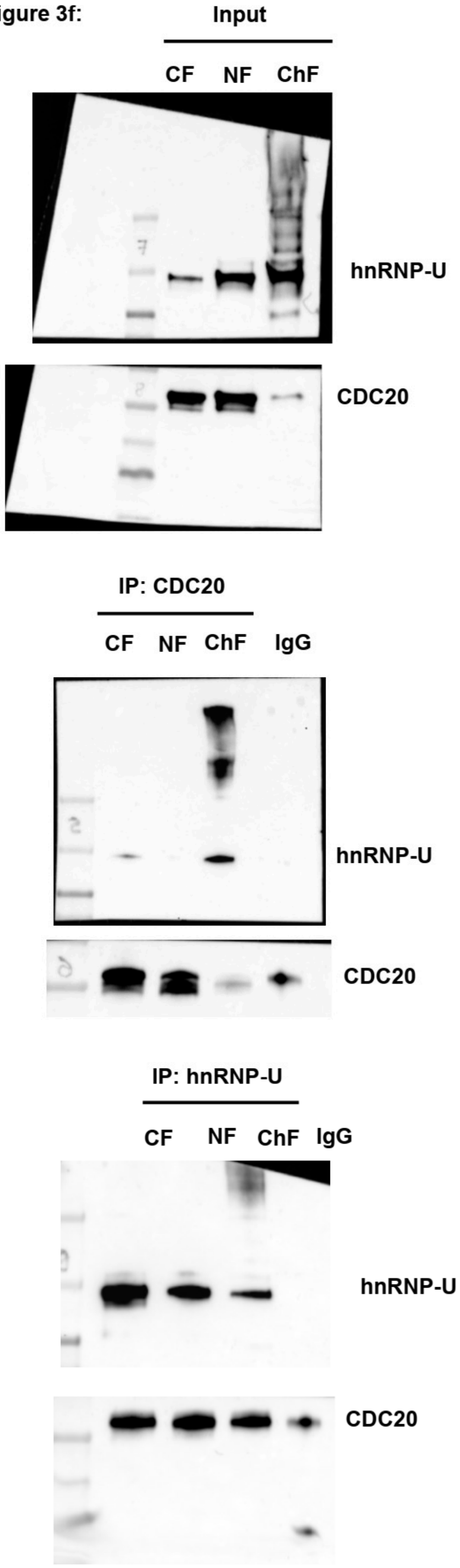

Figure 4a:

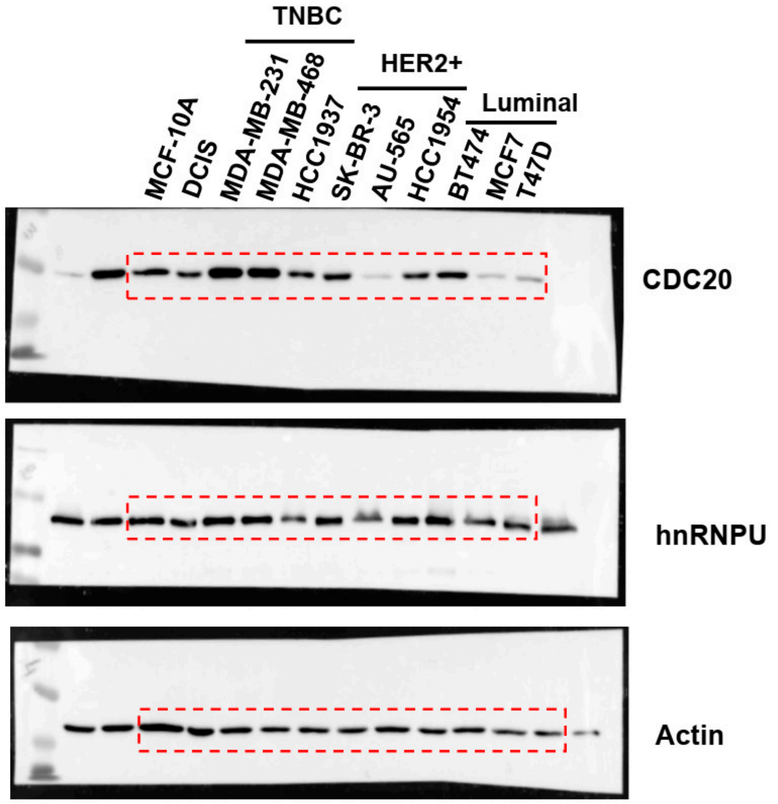

Figure 4g:

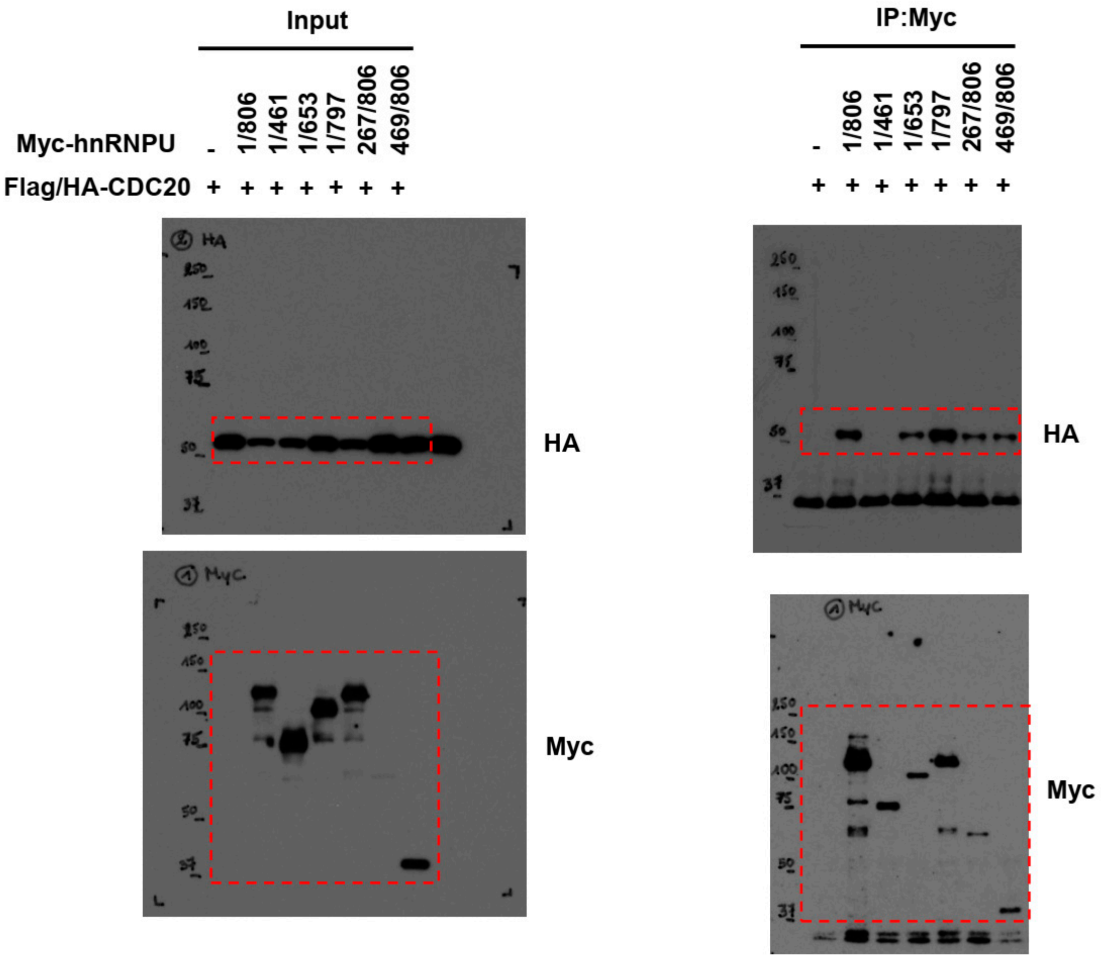

Figure 5a:

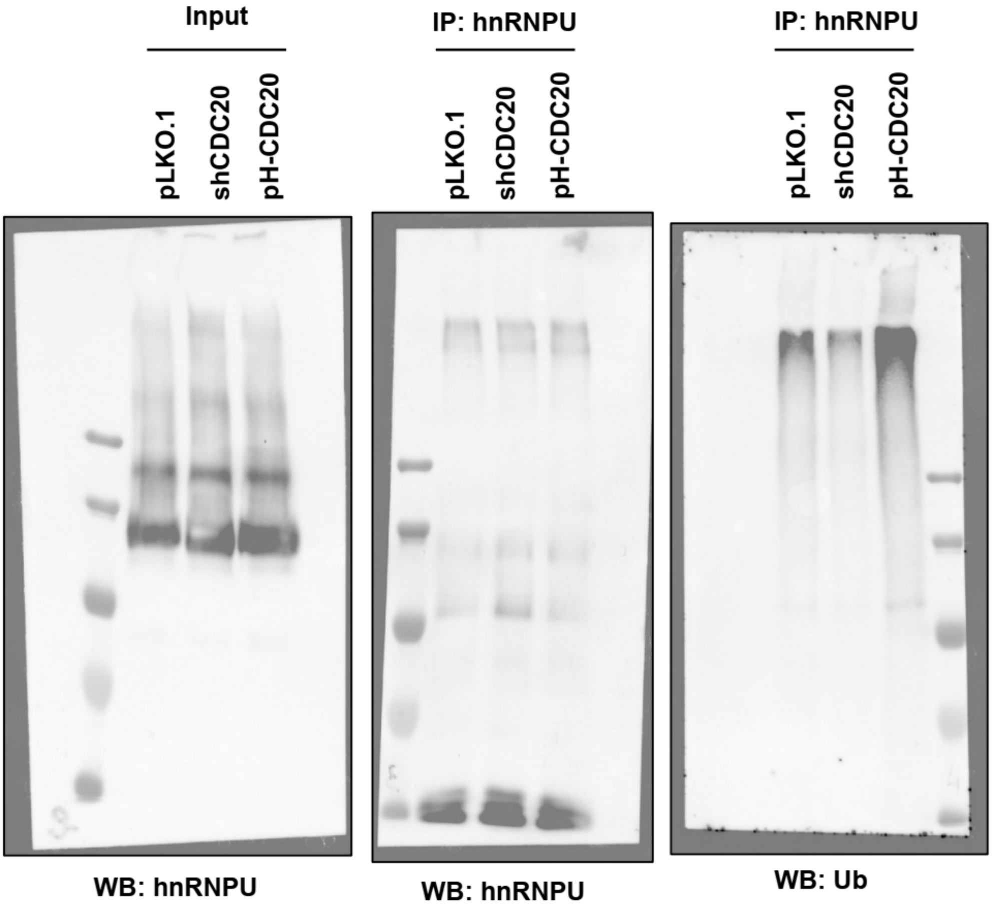

Figure 5b:

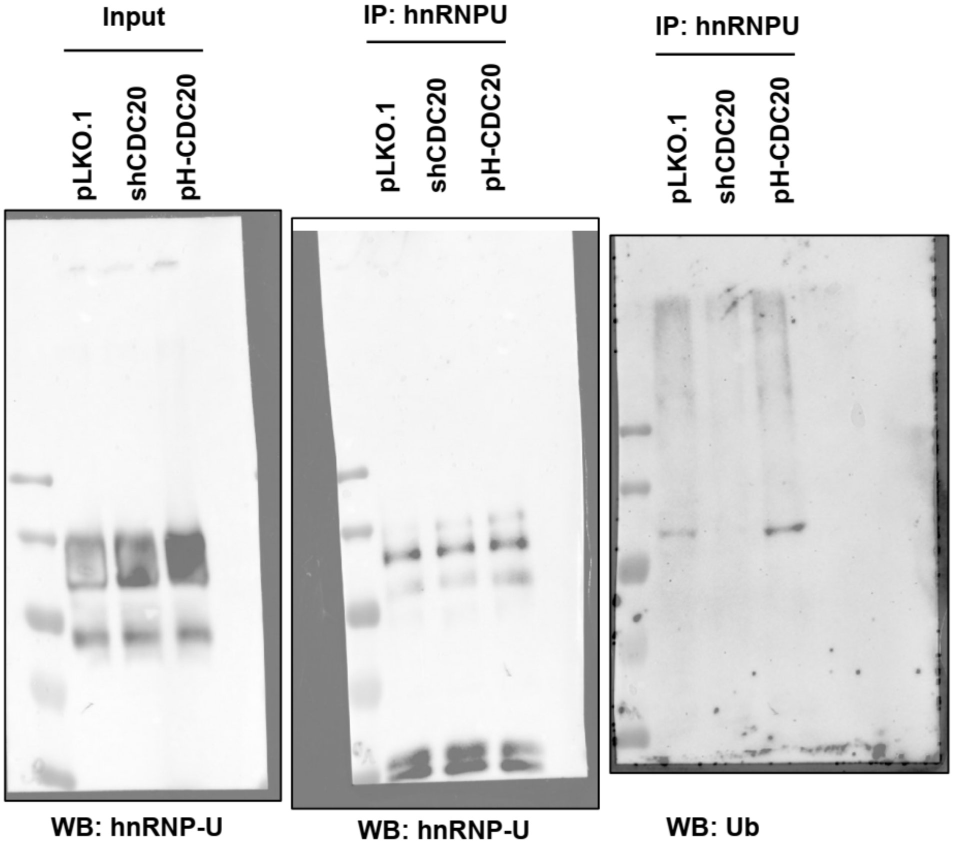

Figure 5c:

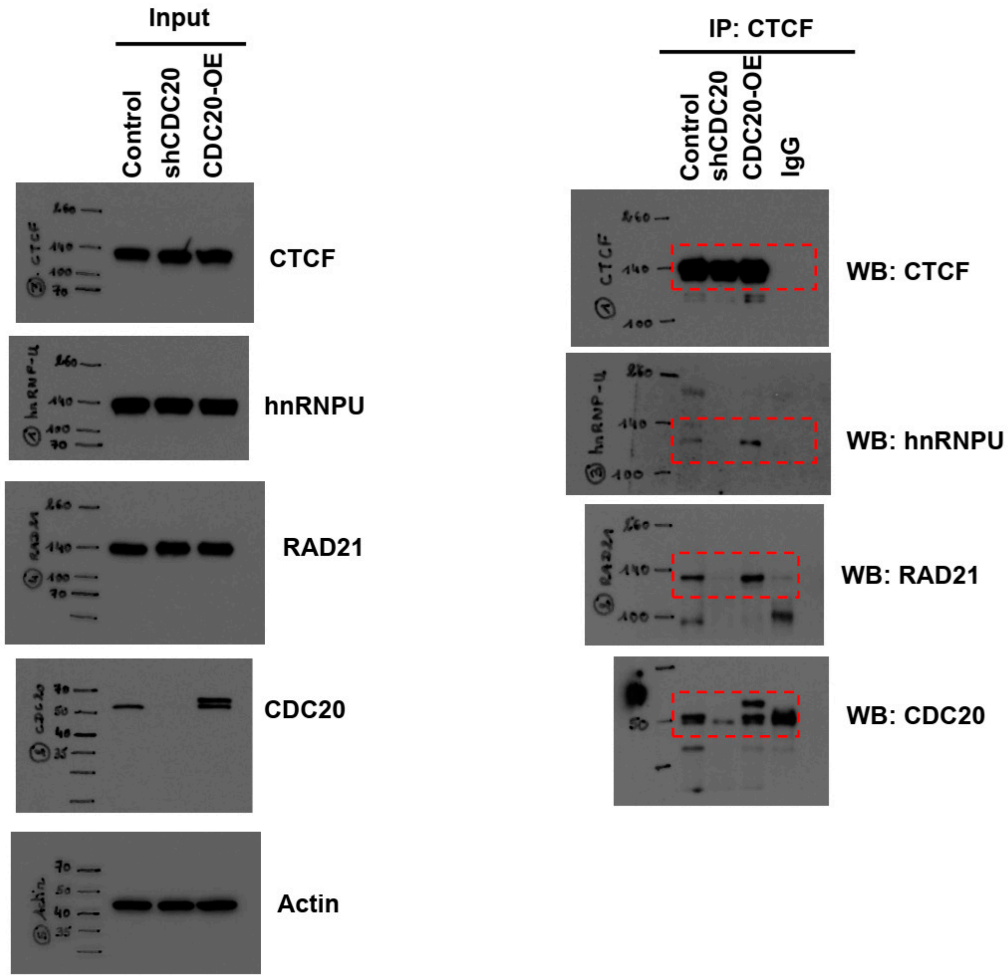

Figure 5d:

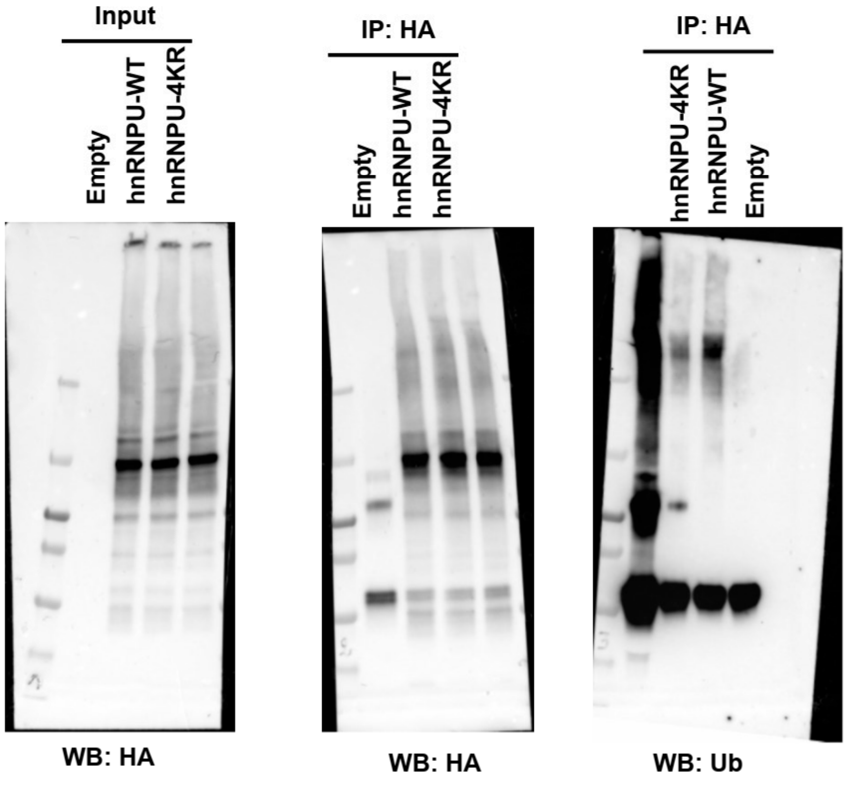

Figure 5e:

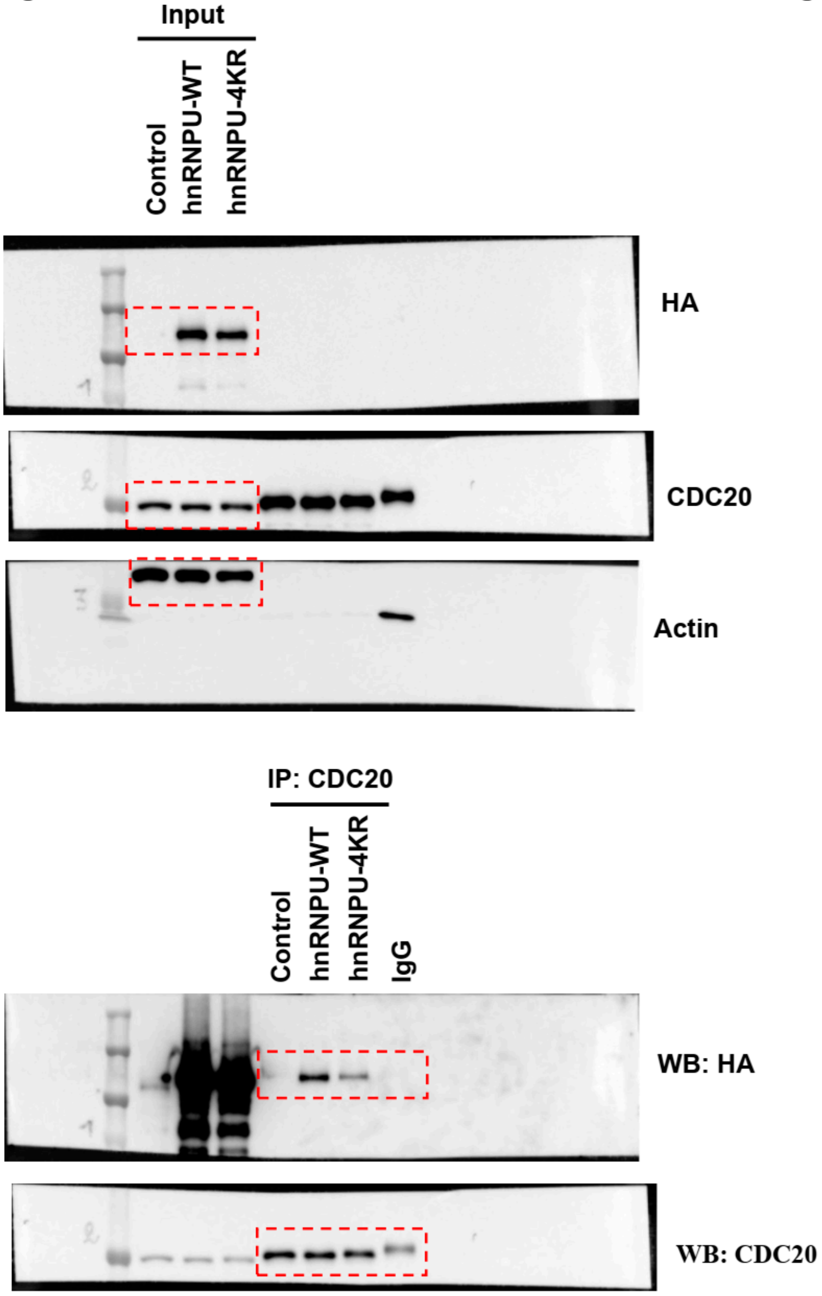

Figure 5f:

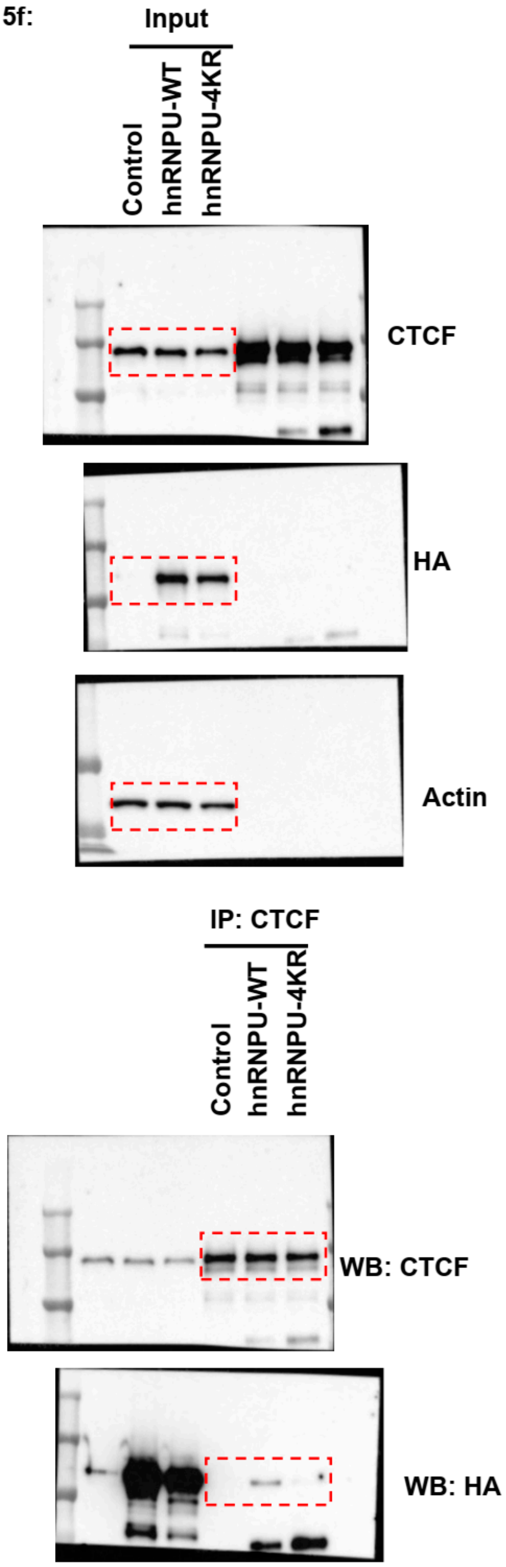

Figure 6e:

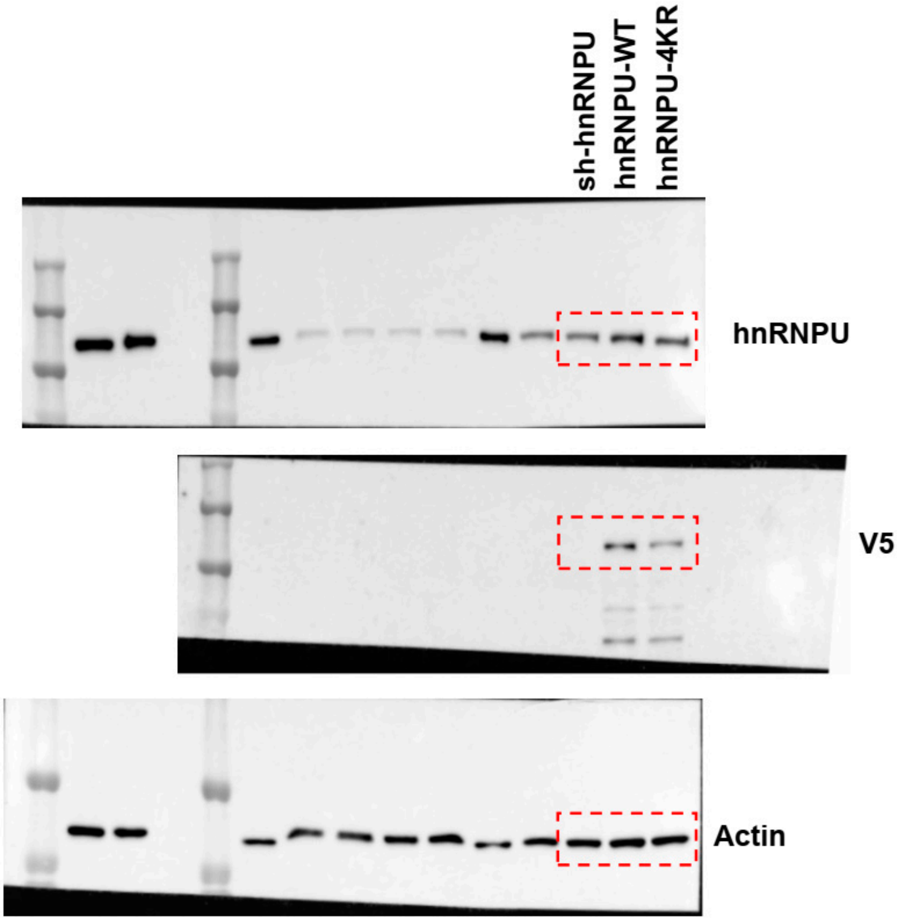

Figure 6i:

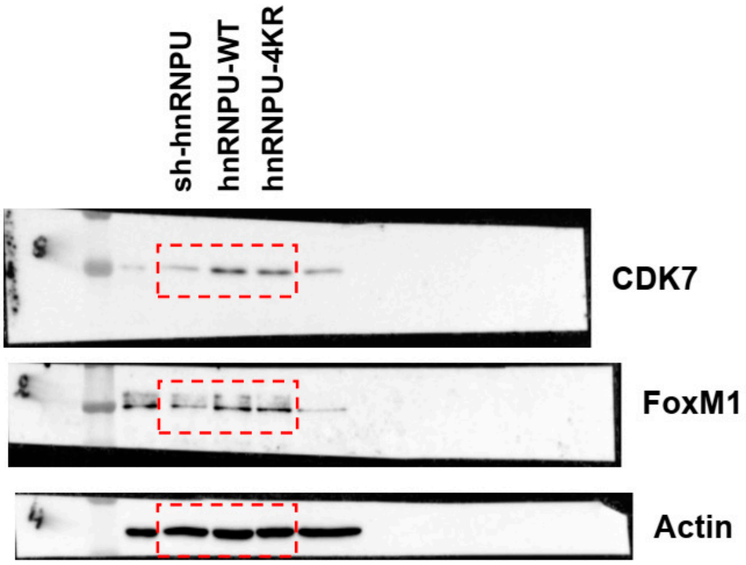

Figure S4a:

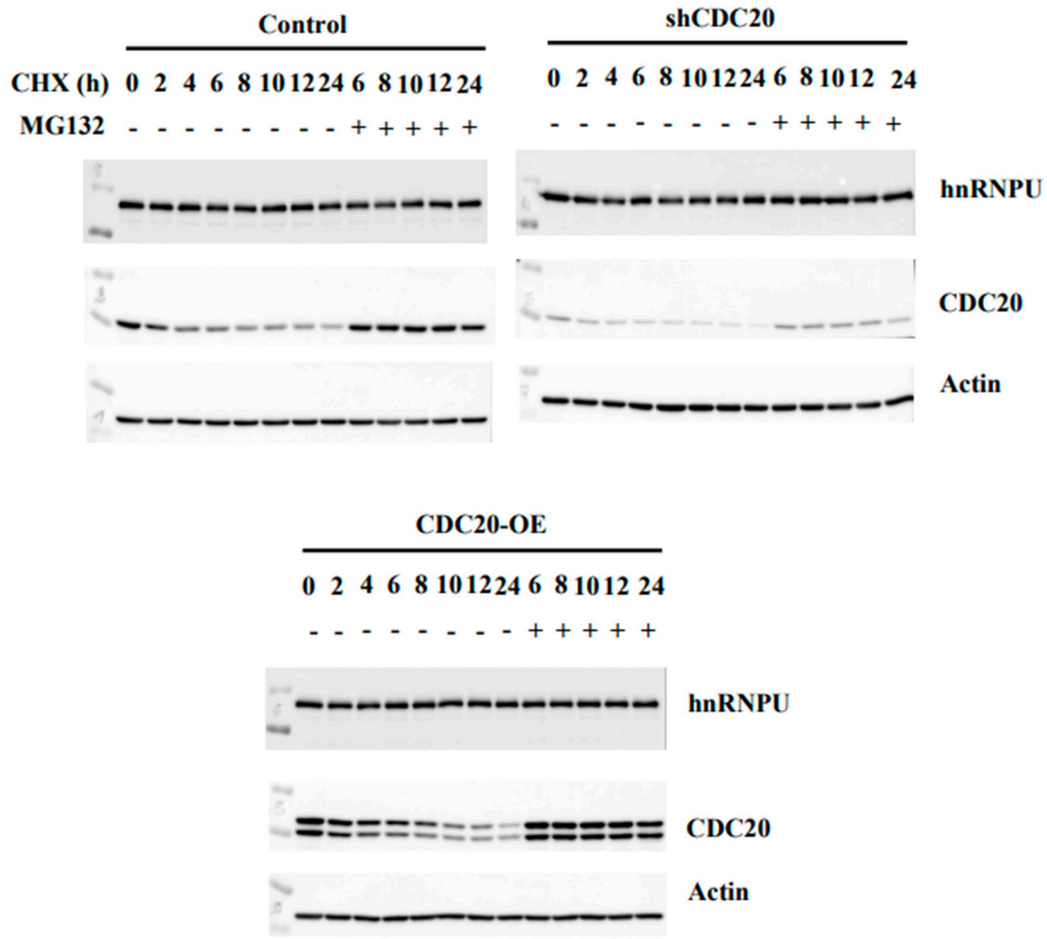

Figure S4b:

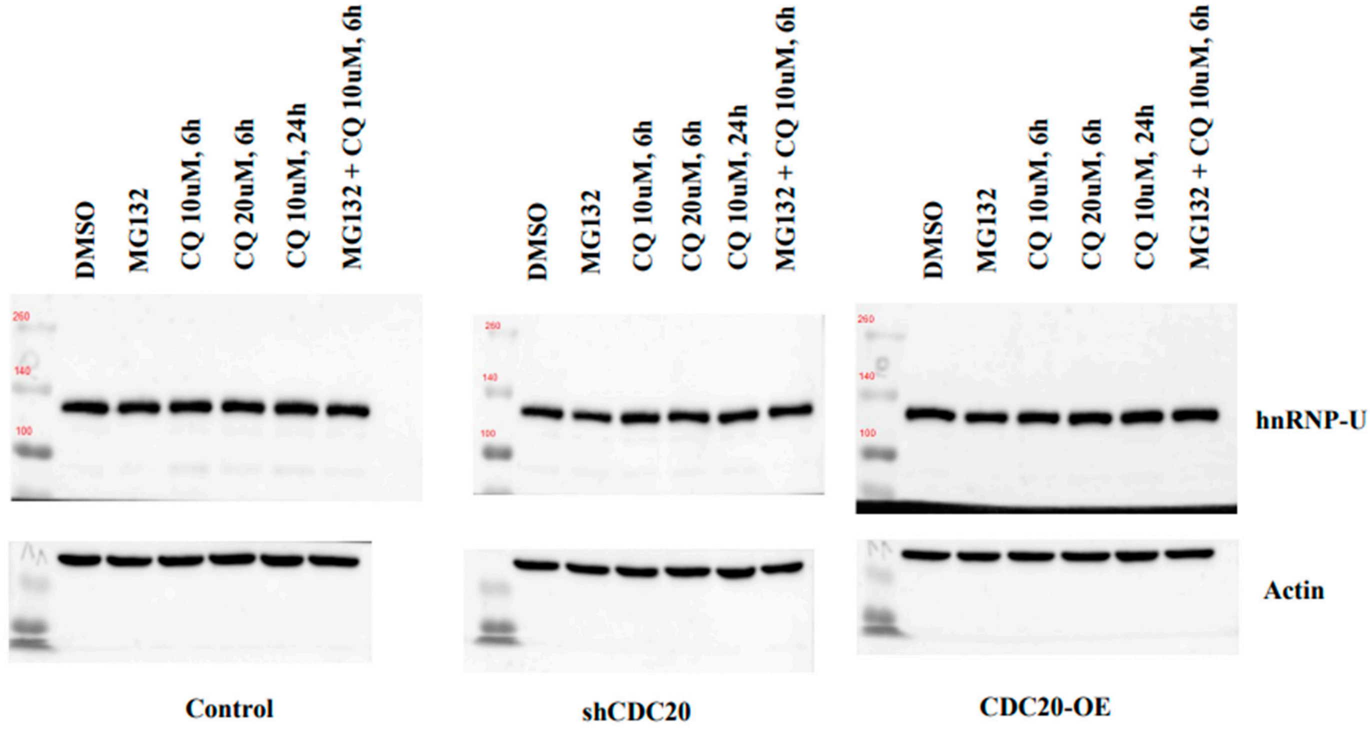

Figure S5a:

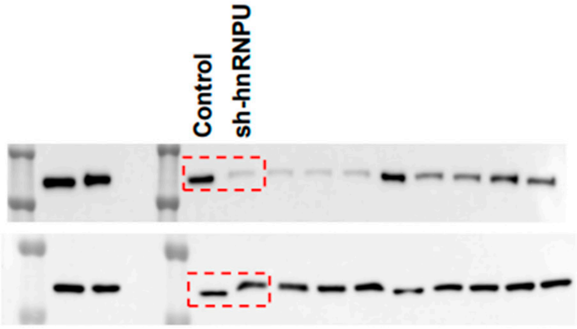

Figure S5c:

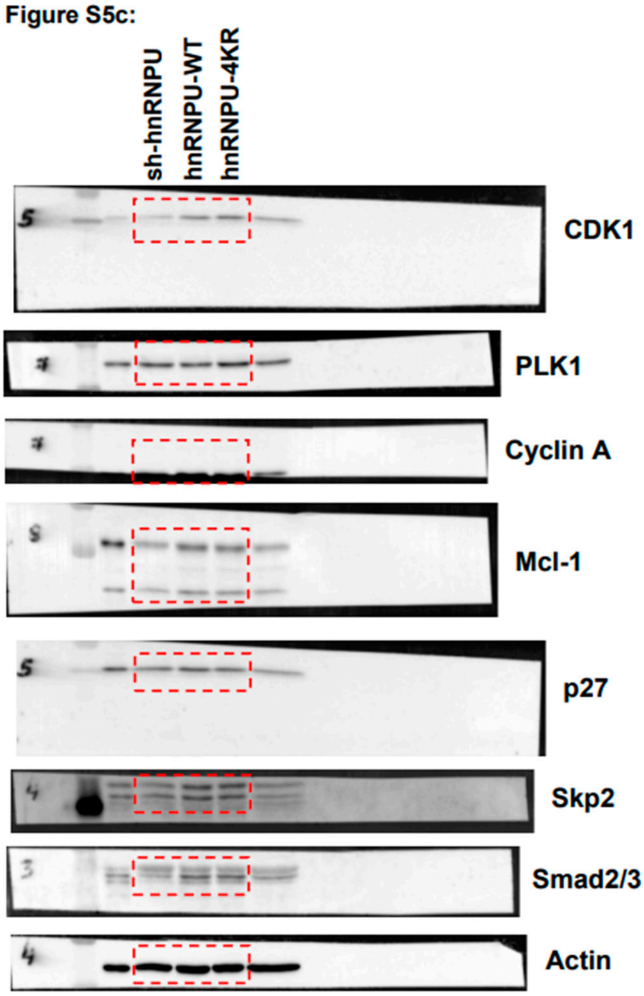

Figure S5b:

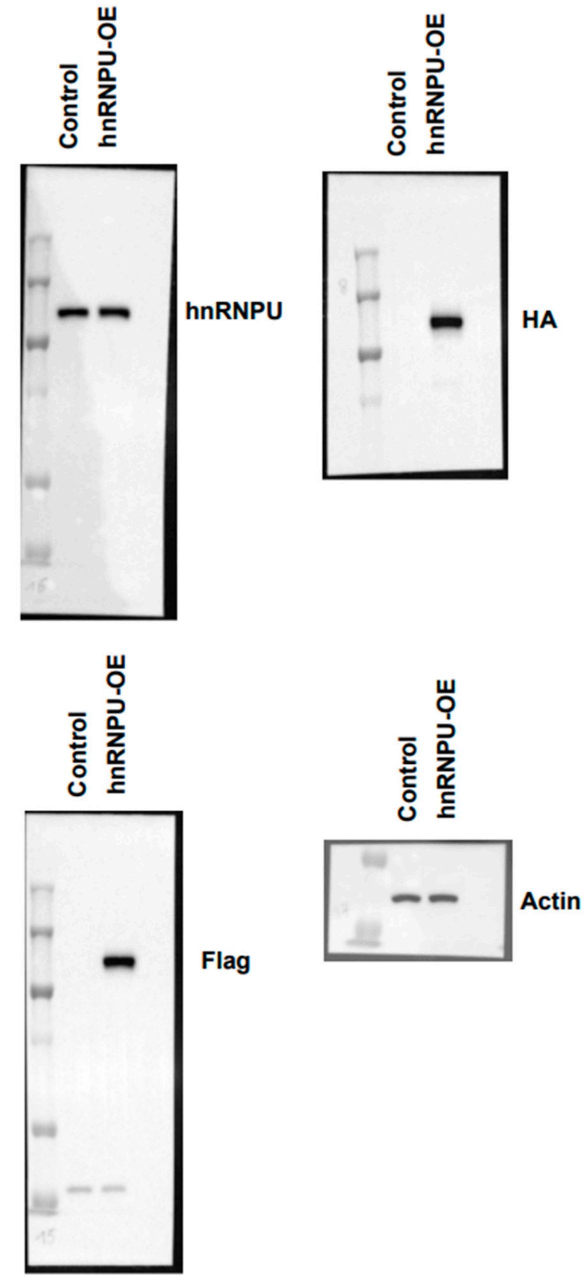

Figure S6. Original uncropped Western blots used in this study.
